# Concordance between In Vitro and In Vivo Relative Toxic Potencies of Diesel Exhaust Particles from Different Biodiesel Blends

**DOI:** 10.3390/toxics12040290

**Published:** 2024-04-16

**Authors:** Subramanian Karthikeyan, Dalibor Breznan, Errol M. Thomson, Erica Blais, Renaud Vincent, Premkumari Kumarathasan

**Affiliations:** 1 Environmental Health Science and Research Bureau, Health Canada, 251, Sir Frederick Banting Driveway, Ottawa, ON K1A 0K9, Canada; dalibor.breznan@hc-sc.gc.ca (D.B.); errol.thomson@hc-sc.gc.ca (E.M.T.); erica.blais@hc-sc.gc.ca (E.B.); 2 Department of Biochemistry, Microbiology & Immunology, Faculty of Medicine, University of Ottawa, Ottawa, ON K1H 8M5, Canada; 3 Interdisciplinary School of Health Sciences, Faculty of Health Sciences, University of Ottawa, Ottawa, ON K1N 6N5, Canada

**Keywords:** biodiesel, diesel exhaust particles, cytotoxicity, intratracheal instillation, inflammation

## Abstract

Diesel exhaust particles (DEPs) contribute to air pollution exposure-related adverse health impacts. Here, we examined in vitro, and in vivo toxicities of DEPs from a Caterpillar C11 heavy-duty diesel engine emissions using ultra-low-sulfur diesel (ULSD) and biodiesel blends (20% *v*/*v*) of canola (B20C), soy (B20S), or tallow–waste fry oil (B20T) in ULSD. The in vitro effects of DEPs (DEP_ULSD_, DEP_B20C_, DEP_B20S_, and DEP_B20T_) in exposed mouse monocyte/macrophage cells (J774A.1) were examined by analyzing the cellular cytotoxicity endpoints (CTB, LDH, and ATP) and secreted proteins. The in vivo effects were assessed in BALB/c mice (n = 6/group) exposed to DEPs (250 µg), carbon black (CB), or saline via intratracheal instillation 24 h post-exposure. Bronchoalveolar lavage fluid (BALF) cell counts, cytokines, lung/heart mRNA, and plasma markers were examined. In vitro cytotoxic potencies (e.g., ATP) and secreted TNF-α were positively correlated (*p* < 0.05) with in vivo inflammatory potency (BALF cytokines, lung/heart mRNA, and plasma markers). Overall, DEP_ULSD_ and DEP_B20C_ appeared to be more potent compared to DEP_B20S_ and DEP_B20T_. These findings suggested that biodiesel blend-derived DEP potencies can be influenced by biodiesel sources, and inflammatory process- was one of the potential underlying toxicity mechanisms. These observations were consistent across in vitro and in vivo exposures, and this work adds value to the health risk analysis of cleaner fuel alternatives.

## 1. Introduction

There are many reports on the links between exposure to particulate air pollutants and adverse cardiac [1,2,3,4,5,6,7,8], pulmonary [9,10], reproductive/pregnancy [11,12,13], and neurological [6,14] outcomes, as well as cancer [15,16,17]. Diesel exhaust particles (DEPs) from traffic emissions are a source of urban air particulate pollution [18] and are considered major contributors to air pollution exposure-related adverse population health effects. There are reports supporting this notion with suggestions of plausible mechanistic pathways for the observed adverse health effects of DEPs [19,20,21,22,23,24].

Biodiesels derived from renewable feedstock may be used as a partial replacement for petroleum-based diesel in automotive applications, which is supported by governmental regulations such as the Clean Fuel Regulations (https://laws-lois.justice.gc.ca/eng/regulations/SOR-2022-140/index.html (accessed on 6 April 2024)), and provincial renewable fuel and low-carbon fuel requirements regulations. A consequence of blending biodiesels with conventional diesel is altered exhaust chemistry. Earlier work on biodiesel [25] showed that biodiesel blends of 20% resulted in reductions of 15% or higher in terms of emissions of particulate matter, carbon monoxide, total hydrocarbons, vapor-phase hydrocarbons from C1 to C12, aldehydes, ketones, selected semi-volatile polycyclic aromatic hydrocarbons (PAHs), and nitroPAHs, with no net effect on the oxides of nitrogen (NOx). There are reports of increased aldehydes, including formaldehyde, acetaldehyde, and acrolein, in biodiesel emissions as opposed to petroleum-based fuel emissions [26,27,28]. The emission of particulate matter (PM) from biodiesel blends on a mass basis was reported to be influenced by the engine starting conditions; furthermore, biodiesel emissions also exhibited altered volatile and semi-volatile PM number and a biodiesel source-dependent increase in NOx emissions [29,30]. Meanwhile, the use of diesel particle filters (DPFs) was shown to decrease PM and CO levels, and hydrocarbons were decreased when using a diesel oxidation catalyst (DOC) + DPF [31,32].

Studies on the toxicological impacts of emissions derived from the combustion of conventional diesel and biodiesel-blended fuels are emerging. Jalava et al. 2010 [33] showed unaltered cytotoxicity and reduced inflammatory potential of DEPs derived from a rapeseed methyl ester biodiesel-blended fuel in comparison to emission particles from conventional diesel. Similarly, Hawley et al., 2014 [34] reported on similar biological responses for a plant-based biodiesel blend (B99) and ultra-low sulfur diesel (ULSD) in human bronchial epithelial cells. Additionally, a report by Rouleau et al., 2013 [35] suggested that air quality and health benefits/costs associated with the use of biodiesel blends and ULSD may be expected to be similar based on modeling with limited data. However, increased toxicity of particulate emissions derived from biodiesel-blended fuels has also been reported. In vitro studies have shown increased cytotoxicity, secreted inflammatory cytokines [36,37], and the production of reactive oxygen species [33,38] after exposure to DEPs derived from biodiesel- blended fuels. Similarly, the exposure of mice to DEPs derived from soy or corn biodiesel-blended fuels via oropharyngeal aspiration resulted in significantly increased inflammatory and oxidative changes in lung tissue [39] or increased sperm DNA fragmentation and the upregulation of inflammatory cytokines in the serum and testes [40]. In an in vitro–in vivo comparative study, Fukagawa et al. (2013) [41] showed increased inflammatory and oxidative stress responses to DEPs derived from 20% *v*/*v* soy biodiesel–ULSD blend when compared to particles derived from ULSD both in vitro in BEAS-2B and THP-1 cell lines and in vivo in C57BL/6 mice. Douki et al. 2018 [42] reported on limited transcriptomic changes in the lungs of rats after repeated exposure to rapeseed oil-derived biodiesel blend and ULSD. Soy-derived biodiesel emissions at a higher PM concentration were reported [43] to be associated with relatively less evidence of pulmonary effects compared to diesel emissions (after 4 weeks of exposure, in vivo). Madden 2016 [44] reviewed the toxicity studies on DEPs derived from the combustion of biodiesel blends and noted the inconsistencies in toxicology findings. Inconsistencies in toxicity findings can arise due to the quality of biodiesel blends and source differences, heterogeneity in exposure conditions/characterization in these studies, the use of different cell types, and the lack of in vitro and in vivo studies carried out with the same biodiesel blends in a systematic manner. 

Therefore, in this work, we examined the relative in vitro and in vivo toxicity characteristics of diesel exhaust particles derived from the combustion of commercial ultra-low sulfur diesel (ULSD) or 20% (*v*/*v*) blends (B20) of biodiesels based on canola oil, soy oil, and tallow–waste fry oil in ULSD used in a heavy-duty diesel engine (on-road technology). These DEPs were examined for in vitro toxicities in mouse monocyte/macrophage cells (J774.A1) and for in vivo toxicities via the intra-tracheal instillation of these DEPs in BALB/c mice, followed by analysis of various biological endpoints to identify any consistency between in vitro and in vivo toxicity findings.

## 2. Materials and Methods

### 2.1. Engine

Emissions were generated using a heavy-duty diesel engine, Caterpillar C11, equipped with the manufacturer’s original diesel oxidation catalyst (DOC) and conforming to 2004 emission standards. The engine specifications and certified emission rates are provided in Table 1. 

Generally, new heavy-duty on-road engines, along with advanced combustion, fueling, and thermal management strategies, are equipped with DPFs and SCR (selective catalyst reduction) systems to meet more stringent emission standards. Future emission standards are based on varying test conditions and are approaching 0.02 g/bhp-hr for NOx and 0.005 g/bhp-hr for PM. 

### 2.2. Fuels

The base test fuel was an ultra-low sulfur diesel (ULSD) procured from a commercial supplier. Unblended, 100% canola methyl ester biodiesel (B100) was procured from Milligan Biotech (Foam Lake, SK, Canada). Soy methyl ester (unblended, B100) was procured from Rothsay Biodiesel (Guelph, ON, Canada). The B100 tallow–waste fry oil was manufactured from a mixed feedstock containing 75% beef tallow and 25% waste fry oil and supplied by Biox Corporation (Oakville, ON, Canada). The biodiesels were blended with ULSD to prepare B20 blends containing 20% *v*/*v* biodiesel methyl ester and 80% *v*/*v* ULSD. The B20 blends of canola methyl ester, soy methyl ester, and tallow–waste fry oil methyl ester are referred to as B20C, B20S and B20T, respectively, in this report. All B100 biodiesels and B20 blends were analyzed by the Alberta Research Council (Edmonton, AB, Canada), operating in compliance with the ISO/IEC 17025 (https://www.iso.org/ISO-IEC-17025-testing-and-calibration-laboratories.html, accessed on 6 April 2024) requirements. Information on the analysis methods used for the characterization of B100 biodiesels and diesel engine emissions related to the fuel types used in this work are provided in Supplementary Tables S1 and S2, and the results of the analysis for B20 biodiesel blends used in the study are provided in Table 2.

### 2.3. Engine Operation for Generation of Test Particles

The engine was operated on a dynamometer. The engine was pre-conditioned by operating at the rated speed and maximum torque for a period of 20 min. During engine operation, the total volume of raw exhaust was transferred from the engine’s exhaust manifold to a constant volume sampling system. The exhaust was diluted with high-efficiency particulate air (HEPA)-filtered ambient air within the dilution tunnel. A continuous flow of diluted exhaust was collected through in-line sampling probes installed in the dilution tunnel and directed to the particulate matter sampling system and gas analyzers. The design of the sampling and analytical systems, as well as engine preparation and operation followed protocols described under the U.S. Code of Federal Regulations (CFR), Title 40, Part 86. For the generation of test particles, the engine was operated in the base OEM configuration (with DOC), at 25% or 50% loads in a steady-state 1200 rpm using ULSD, B20C, B20S, or B20T. The particulate matter samples were collected using Zefluor^®^ 8 “×10” rectangular polytetrafluoroethylene (PTFE) filters (Pall Life Sciences, Port Washington, NY, USA) for toxicity testing. The engine operation conditions and emission rates are given in Table 3.

### 2.4. Emission Characterization

Emissions of carbon monoxide (CO), carbon dioxide (CO_2_), oxides of nitrogen (NOx), and total hydrocarbons (THCs) were continuously monitored in the emission stream. Particles were collected using 70 mm EmFab^®^ filters (Pall Life Sciences, Port Washington, NY, USA) for gravimetry. Particulate filters were handled according to the procedure described in 40 CFR 86.1339-90 Particulate filter handling and weighing. Fuel consumption was calculated using an industry-standard carbon balance equation. The details of the analytical methods and instrumentation used in the characterization of emissions are provided in Supplementary Table S2.

### 2.5. Extraction of Particles

Pre-weighed sections of Zefluor^®^ PTFE filters (VWR International, Mississauga, ON, Canada) containing diesel exhaust particles were placed in a 50 mL falcon tube (VWR, Mississauga, ON, Canada) and wetted with 2 mL of 100% ethanol (Sigma Chemical Company, MO, USA) for 60 s. The filters were then immersed in sterile, deionized water and sonicated in an ice-cold water bath for 30 min. Following sonication, the filters were removed, dried, and conditioned to calculate mass recovery. The particle extract was transferred into a pre-weighed, pre-siliconized lyophilization flask and lyophilized using Freezone lyophilizer (LabConco, Kansas City, MO, USA) according to the manufacturer’s instructions. The lyophilized material was then suspended in a small volume of deionized water and aliquoted in pre-siliconized and pre-weighed amber glass tubes and lyophilized again. The samples were then frozen at −80 °C. The emission particles derived from the combustion of ULSD, B20C, B20S, and B20T are referred to below as DEP_ULSD_, DEP_B20C_, DEP_B20S_ and DEP_B20T_, respectively.

### 2.6. Preparation of Particle Suspensions

Lyophilized diesel exhaust particles were suspended in a particle preparation buffer (25 μg/mL of Tween-80 in 0.19% saline solution) at a concentration of 10 mg mL^−1^, as described previously [45]. The particle suspensions were vortexed for 30 s and then sonicated for 20 min in ice-cold water in a water bath sonicator. The particles were then homogenized with 25 strokes of a homogenizer. In order to obtain a sufficient mass of particles to conduct both in vitro and in vivo toxicity testing experiments, the particles derived from engine runs at 25% and 50% loads were combined at a 2:1 mass ratio. This was rationalized based on the findings shown in Table 3. Even though engine loads of 25% vs. 50% were associated with different PM masses, at the same engine load across all fuels, the PM masses were similar and enabled the pooling of particles obtained from the different loads for the same fuel type, as noted above. The particles were aliquoted in O-ring-capped micro-centrifuge tubes and heated in a water bath at 56 °C for 30 min for sterilization. After cooling to room temperature, the samples were stored at −40 °C until testing. 

### 2.7. In Vitro Exposure

#### 2.7.1. Cell Culture

The J774A.1 cells were propagated in Dulbecco’s Modified Eagle’s Medium (DMEM; Fisher Scientific) containing phenol red and 4.5 g/L glucose and supplemented with FBS (10% *v*/*v*, non-heat inactivated; Fisher Scientific) and Pen Strep (100 U/mL penicillin G, 100 µg/mL streptomycin; Invitrogen, Burlington, ON, Canada) (referred below as complete DMEM) in 75 cm^2^ tissue culture flasks (Corning, NY, USA). Unless stated otherwise, all of the cell incubations were at 5% CO_2_ and 95% relative humidity. In preparation for cytotoxicity bioassays, the cells were recovered by scrapping the monolayers in complete DMEM devoid of phenol red. Cell suspensions in complete DMEM of 4 × 10^5^ cells/mL were seeded in 96-well black-walled clear-bottom cell culture plates (BD Biosciences, Mississauga, ON, Canada) at 100 µL/well (4 × 10^4^ cells/well, 12 × 10^4^ cells/cm^2^) and the plates were incubated at 37 °C for 24 h. 

#### 2.7.2. DEP Exposure

Lyophilized DEP suspensions were thawed at room temperature and sonicated for 20 min in an ice-cold ultrasonic water bath and diluted in complete DMEM (devoid of FBS. phenol red). The dilute particle suspensions were sonicated for 5 min in an ice-cold, ultrasonic water bath prior to dosing the cells in the 96-well plates. Cells in 100 µL cell culture medium with 10% FBS were dosed with a 100 µL particle suspension in culture medium (with no FBS) to have final doses 0, 10, 30, 100, 300 µg/cm^2^ (final volume of 200 µL/well and the final concentration of FBS was 5%). 

#### 2.7.3. Cytotoxicity Analyses

J774A.1 cells were incubated for an additional 24 h post-exposure to DEPs before the assessment of cytotoxicity using an integrated bioassay that combined assays of cellular redox status (CellTiter Blue^®^ Assay), energy metabolism (ATP assay), and membrane integrity (intracellular lactate dehydrogenase (LDH) release assay), as described by Kumarathasan et al., 2015 [46]. CellTiter-Blue^®^ and the Cytotox ^®^ kit for LDH release assay were purchased from Promega Corporation (Madison, WI, USA). The ATP detection kit was procured from Lonza Rockland Corporation (Rockland, ME, USA). All of the exposure experiments were repeated three times for each cell line, with two technical replicates/experiments. 

#### 2.7.4. Cytokine Secretion

The cell culture supernatants of J774.A1 were assessed using a 23-plex mouse multiplex cytokine assay panel (Bio-Rad Laboratories (Canada) Ltd., Mississauga, ON, Canada) for secretion of IL-1α, IL-1β, IL-2, IL-3, IL-4, IL-5, IL-6, IL-9, IL-10, IL-12 (p40), IL-12 (p70), IL-13, IL-17A, eotaxin, G-CSF, GM-CSF, IFN-γ, KC, MCP-1 (MCAF), MIP-1α, MIP-1β, RANTES, and TNF-α following the manufacturer’s protocols. Analyses were conducted on a Bio-Plex 200 multiplex luminescence assay system (Bio-Rad Laboratories Canada Ltd., Mississauga, ON, Canada). 

### 2.8. In Vivo Exposure 

#### 2.8.1. Animals

Specific-pathogen-free BALB/c mice (male, weight 26.4 ± 0.2 g, mean ± standard error) obtained from Charles River (St Constant, QC, Canada) were housed in individual Plexiglass cages on wood-chip bedding and were held under a 12:12 h dark: light cycle. Food and water were provided ad libitum. Animals were received and housed in the animal care facilities of Health Canada in Ottawa. All of the experimental protocols were reviewed and approved by the Animal Care Committee of Health Canada. 

#### 2.8.2. Intratracheal Instillation

The animals were administered 250 µg of DEP or carbon black (n = 6) via a single intratracheal instillation, in line with previous reports. Carbon black was used as a reference particle as it is a widely accepted model particle in diesel studies. A group of animals were instilled with saline as the vehicle control (n = 6). The instillation of the experimental animals was conducted over a two-day period. In order to eliminate bias from the day of instillation, half the number of animals from each exposure group were instilled on day one and the other half of animals were instilled on day two. Before DEP instillation, the animals were anaesthetized via isoflurane inhalation and placed on their backs on a 40° slope. With the tongue pressed towards the lower jaw by a small sterile spatula, a 24-gauge catheter with a shortened needle was placed in the trachea. A sensitive pressure transducer was connected to the catheter to confirm the positioning of the instillation needle in the trachea. Particle preparations to be instilled were suspended by placing in an ultrasonic water bath for 5 min, followed by rigorous pipetting just prior to the removal of an aliquot for instillation. In a 250 µL SGE glass syringe (Fisher Scientific, Ottawa, ON, Canada), a sandwich of 50 µL of air, 50 µL of DEP suspension or saline (vehicle), and 150 µL of air to be delivered into the lungs in this order was prepared. Prior to instillation, the pressure transducer was disconnected from the catheter. The tip of a glass syringe loaded with a particle suspension or saline was inserted into the catheter, and the material was delivered with a stroke of the plunger. Immediately after instillation, the catheter was removed, and the mouse was held head up momentarily to ensure the delivered material remained in the lungs. After the animals recovered from anesthesia, they were transferred back to their cages for recovery. 

#### 2.8.3. Biological Samples

Following a 24 h recovery period after intratracheal instillation, the animals were anaesthetized via isofluorane. The blood was withdrawn via cardiac puncture into vacutainer tubes containing the sodium salt of EDTA at 10 mg/mL and PMSF at 1.7 mg/mL, mixed gently, and placed on ice. The diaphragm was then punctured, to expose the trachea, which was then cannulated. The lungs were filled via the intratracheal delivery of filter-sterilized, warm saline (0.9%, 37 °C) at 30mL/kg body weight [47]. The lungs were massaged gently by rubbing the thoracic cage. Saline was aspirated and reinjected twice more, and the primary bronchoalveolar lavage (BAL) was collected in a 15 mL centrifuge tube kept on ice. Secondary lavages were obtained with additional volumes of saline (5 mL/animal), three times, to increase the yield of lavage cells. The lavage fluids were centrifuged (1500 rpm for 10 min at 4 °C) to separate the cells from the supernatants. The cell pellets from both primary and secondary lavages were combined to recover the total BAL cells. Primary lavage supernatants were used to analyze biochemical endpoints. Secondary lavage supernatants were discarded. Whole-blood samples were centrifuged at 2000 rpm for 10 min at 4 °C to obtain plasma. Plasma aliquots were frozen at −80 °C. Lung and heart tissues were collected, flash-frozen in liquid nitrogen, and stored at −80 °C for reverse transcriptase-PCR analyses.

#### 2.8.4. Cytology

Lung BAL cells were counted using a Coulter Multisizer II (Coulter Canada, Ville St-Laurent, QC, Canada), and differential cell counts were obtained from cytospin preparations using Wright stain and following standard procedures [48]. 

#### 2.8.5. Cytokines

Levels of interleukin (IL)-1α, IL-1β, IL-2, IL-3, IL-4, IL-5, IL-6, IL-9, IL-10, Il-12 (p40), IL-12 (p70), IL-13, IL-17, eotaxin, granulocyte–macrophage colony-stimulating factor (G-CSF), GM-CSF, interferon (IFN)-γ, growth-related oncogene/keratinocyte chemoattractant (GRO/KC), monocyte chemoattractant protein-1 (MCP-1), macrophage inflammatory protein (MIP)-1α, MIP-1β, regulated on activation and normal T cell expressed and secreted chemokine (RANTES), and tumor necrosis factor-α (TNF-α) in the BAL fluid and plasma were analyzed using a 23-plex cytokine assay kit (Millipore Corporation, Billerica, MA, USA). The plasma levels of selectin, matrix metalloproteinase (MMP)-9, soluble intracellular adhesion molecule (sICAM), soluble vascular cell adhesion molecule (sVCAM), plasminogen activator inhibitor (PAI), apolipoprotein A1 (apoA1), apolipoprotein E (apoE), fibrinogen, and adiponectin were analyzed using cardiovascular multiplex cytokine assay kits (Millipore Corporation). The analyses were conducted according to the manufacturer’s instructions using a Bio-Plex 200 multiplex luminescence assay system (Bio-Rad Laboratories Canada Ltd.).

#### 2.8.6. Gene Expression Analyses

Lung and heart samples were homogenized in TRIzol^®^ reagent (Invitrogen Canada, Inc., Burlington, ON, Canada), and the total RNA was isolated according to the manufacturer’s instructions. The RNA was quantified using the RiboGreen RNA Quantitation Reagent and Kit (Molecular Probes, Eugene, OR, USA), and the total RNA was reverse transcribed using MuLV reverse transcriptase and random hexamers (Applied Biosystems, Mississauga, ON, Canada), according to the manufacturer’s instructions. Primers for TATA Binding Protein, oxyguanine glycosylase (OGG)-1, IL-1β, IL-6, endothelin (ET)-1, inducible nitric oxide synthase (iNOS), endothelial nitric oxide synthase (eNOS), cytochrome P450, family 1, subfamily A, polypeptide 1 (CYP1A1), metallothionein 2A (MT2A), prostaglandin–endoperoxide synthase 2 (PTGS2), heme oxygenase-1 (HO-1), and hypoxia-inducible factor (HIF)-3α were obtained from Thomson et al. 2013 [49] or designed and validated to produce amplicons with an optimal annealing temperature of 60 °C (Supplementary Table S3). Real-time PCR was performed using 96-well plates in a spectrofluorometric thermal cycler (Lightcycler 480, Roche Diagnostics Canada, Laval, QC, Canada) using iQ SYBR Green Supermix (Bio-Rad Laboratories [Canada] Ltd.), as previously described by Thomson et al., 2013 [49]. A melt curve was conducted following each run to verify the product’s purity. The expression was calculated relative to peptidylprolyl isomerase A expression using the delta–delta Ct method and expressed relative to time-matched controls.

#### 2.8.7. Potency Calculations

Data for in vitro cytotoxicity and for the secretion of cytokines and chemokines in J774A.1 cells were normalized to control values to obtain the fold effect for each particle dose. Potency (β) was derived from the equation below.

Fold Effect = (Dose + 1)^β^

where β is the slope of the dose–response relationship for a given endpoint [50]. The dose–response data were fitted using CurveExpert v1.3 (D. Hyams, Hixson, TN, USA) to calculate cytotoxic potency and potencies based on cytokine responses in J774.A1 cells. Because only a single dose of particles was instilled in vivo, the in vivo biological potency was calculated as the ratio of the biological effect of a DEP to that of a saline vehicle control for a given bioassay (i.e., fold change).

### 2.9. Statistical Analyses

For in vitro exposure, cytotoxicity and cytokine secretion data were analyzed via two-way ANOVA with *PM* (DEP_ULSD_, DEP_B20C_, DEP_B20S_, and DEP_B20T_) and *DOSE* (0, 10, 30, 100, and 300 µg/cm^2^) as factors. Datasets not meeting the assumptions of normality and equal variance for ANOVA were subjected to transformation (e.g., log_10_, ln, inverse or square root or ranks) prior to analyses. 

In vivo exposure responses were analyzed through the use of one-way ANOVA for differences between the groups of DEP_ULSD_, DEP_B20C_, DEP_B20S_, DEP_B20T,_ CB, and saline-exposed animals. Datasets that did not meet the assumptions of normality and equal variance were assessed via Kruskal–Wallis one-way analysis of variance on ranks. For both one-way and two-way ANOVAs, pairwise multiple comparisons were carried out using Tukey’s test to elucidate the pattern of significant effects (α = 0.05). All of the analyses were conducted using SigmaPlot, version 12 (Systat Software, Inc., San Jose, CA, USA). Pearson product moment correlations between in vitro and in vivo biological potencies were calculated using SPSS version 15 (IBM Corporation, New York, NY, USA). 

## 3. Results

### 3.1. In Vitro Effects

#### 3.1.1. Cytotoxicity

**Figure 1:**
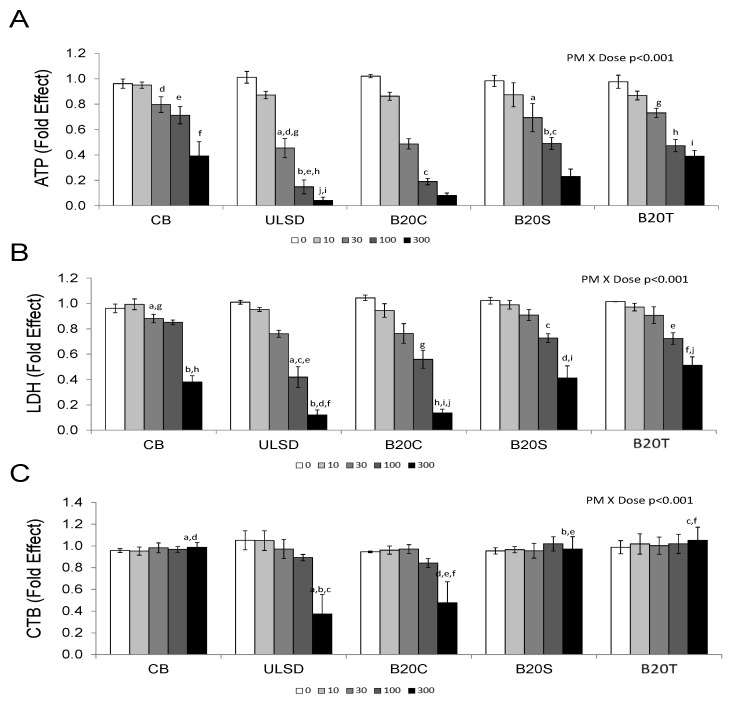
The figure shows the impact of biodiesel feedstock on the cytotoxicity of diesel exhaust particles assessed in the J774.A1 macrophage cell line. (Values are mean ± SEM; n = 3 experiments; similar notations (letters) are significantly different (*p* < 0.05) from each other). (**A**) Intracellular ATP content, Two-way ANOVA, *PM* × *DOSE*, *p* < 0.001. (**B**) Intracellular LDH content, Two-way ANOVA, *PM* × *DOSE*, *p* < 0.001. (**C**) Resazurin reduction, two-way ANOVA, *PM* × *DOSE*, *p* < 0.001.

Biodiesel feedstock impacted the cytotoxicity of DEP (Figure 1). Higher doses (100 and 300 µg/cm^2^) of DEP_ULSD_ and DEP_B20C_ were relatively more cytotoxic than the same doses of CB, DEP_B20S_ or DEP_B20T_ based on all cytotoxicity assays (two-way ANOVA, *PM* × *DOSE* interaction, *p* < 0.001; Figure 1). 

#### 3.1.2. In Vitro Cytokine Secretion

**Figure 2:**
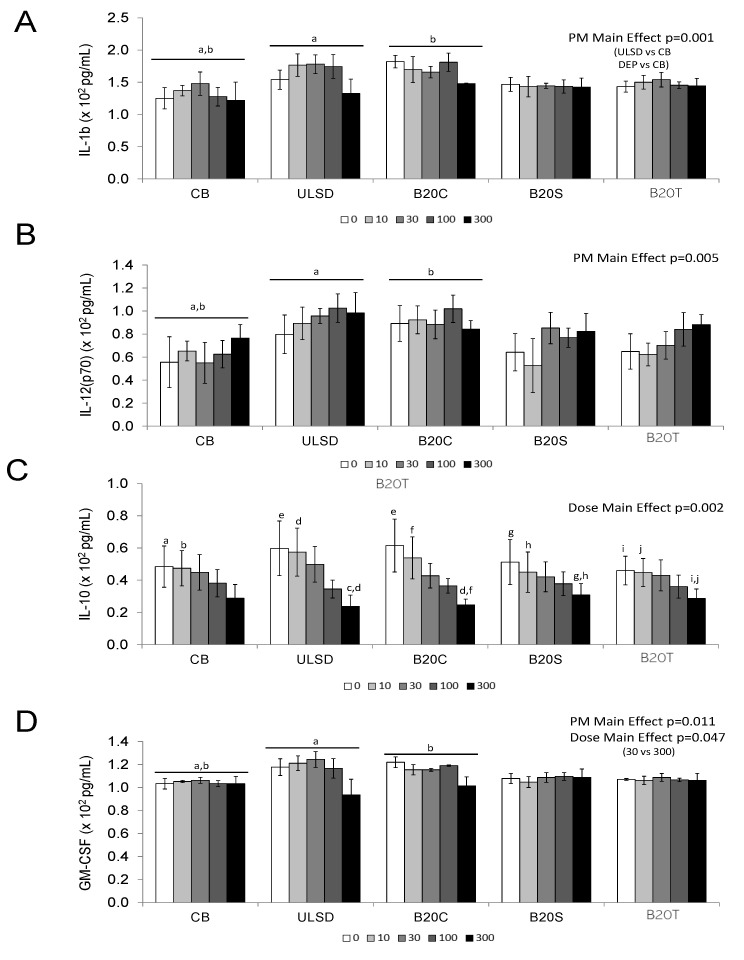

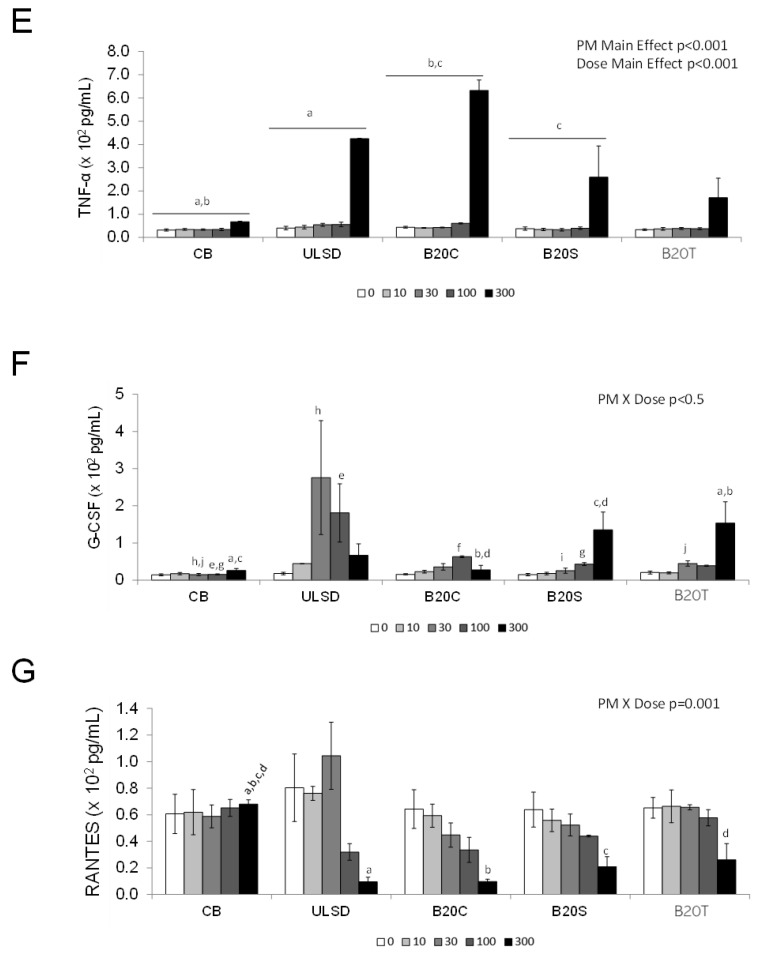
The figure shows in vitro cytokine secretion in J774.A1 in response to exposure to diesel exhaust particles. (Values are mean ± SEM; n = 3 experiments; similar notations (letters) are significantly different (*p* < 0.05) from each other) (**A**) IL1-β, 2-way ANOVA, PM main effect, *p* = 0.001. (**B**) IL-12 (p70), 2-way ANOVA, PM main effect, *p* = 0.005. (**C**) IL-10, Two-way ANOVA, DOSE Main Effect, *p* = 0.002. (**D**) GM-CSF, *PM* Main Effect, *p* = 0.011. *DOSE* Main Effect, *p* = 0.047. (**E**) TNF-α, *PM* main effect, *p* < 0.001; *DOSE* Main Effect, *p* < 0.001. (**F**) G-CSF, 2-way ANOVA, *PM* × *Dose* interaction, *p* <0.05 (**G**) RANTES, 2-way ANOVA, *PM* × *Dose* interaction, *p* < 0.001.

Secretion of inflammatory cytokines and chemokines by J774A.1 cells in response to DEP exposure was impacted by biodiesel feedstock. Two-way ANOVA results identified PM main effect for IL-1β and IL-12 (p70) (*p* < 0.05, Figure 2A,B), whereas Dose main effect was noticed with IL-10 (*p* < 0.05, Figure 2C). 

Furthermore, secreted GM-CSF and TNF-α levels revealed the main effects of PM and dose (*p* < 0.05; Figure 2D,E), with all particles exhibiting increased TNF-α levels at 300 µg/cm^2^ dose compared to the vehicle control and DEP_ULSD_ and DEP_B20C_ showed relatively higher levels of TNF-α at this dose compared to the other particles tested in this study. Two-way ANOVA analysis results for secreted G-CSF and RANTES identified Particle × Dose interaction (*p* < 0.05, Figure 2F,G). The secretion of RANTES decreased with dose, with relatively larger declines noted after exposure to the highest doses of DEP_ULSD_ or DEP_B20C_.

### 3.2. In Vivo Effects

#### 3.2.1. BAL Neutrophil Counts

**Figure 3:**
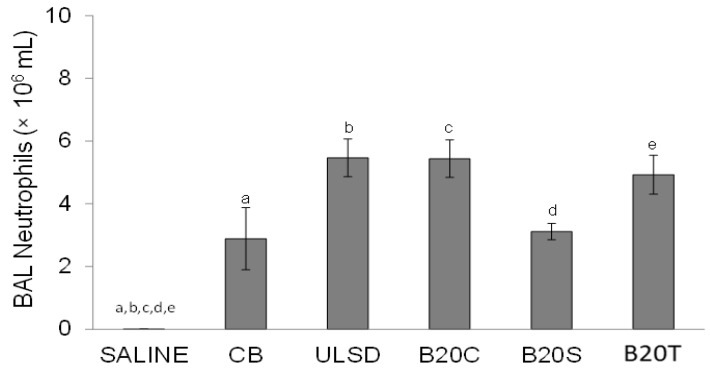
The figure shows the BAL neutrophil profiles following the instillation of DEPs (values are mean ± SEM; n = 6/group; One-way ANOVA results: similar notations (letters) are significantly different (*p* < 0.05) from each other).

All particles, including CB, caused a significant increase in the number of lung lavage neutrophils compared to saline (the vehicle control) 24 h after the intratracheal instillation of particles in these mice (one-way ANOVA, *p* < 0.05; Figure 3), with increasing trends noted in the animals instilled with DEP_ULSD_, DEP_B20C_, and DEP_B20T_ compared to the other particles. 

#### 3.2.2. BAL Cytokines

**Figure 4:**
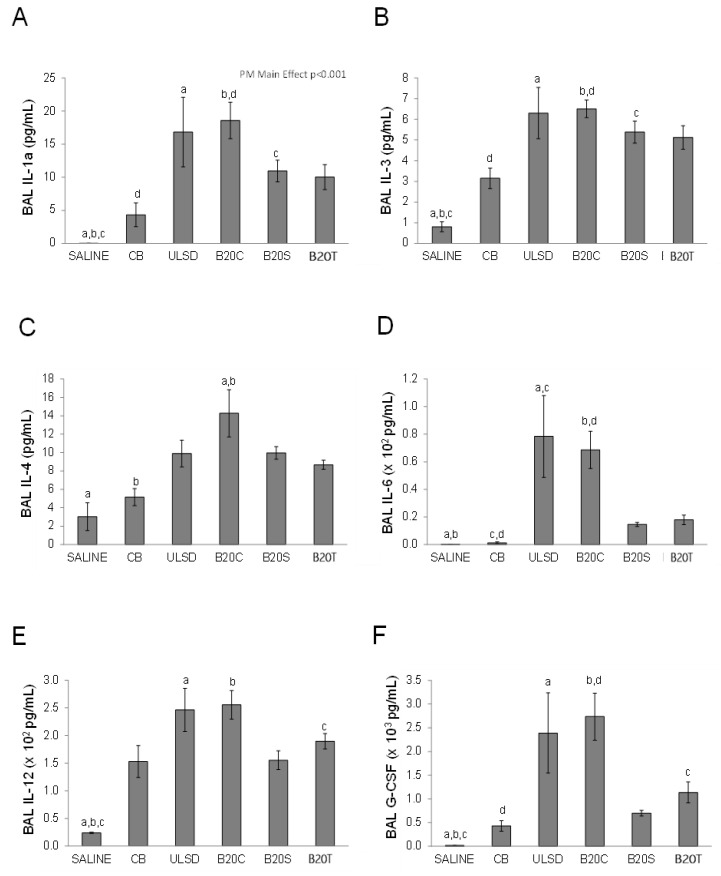

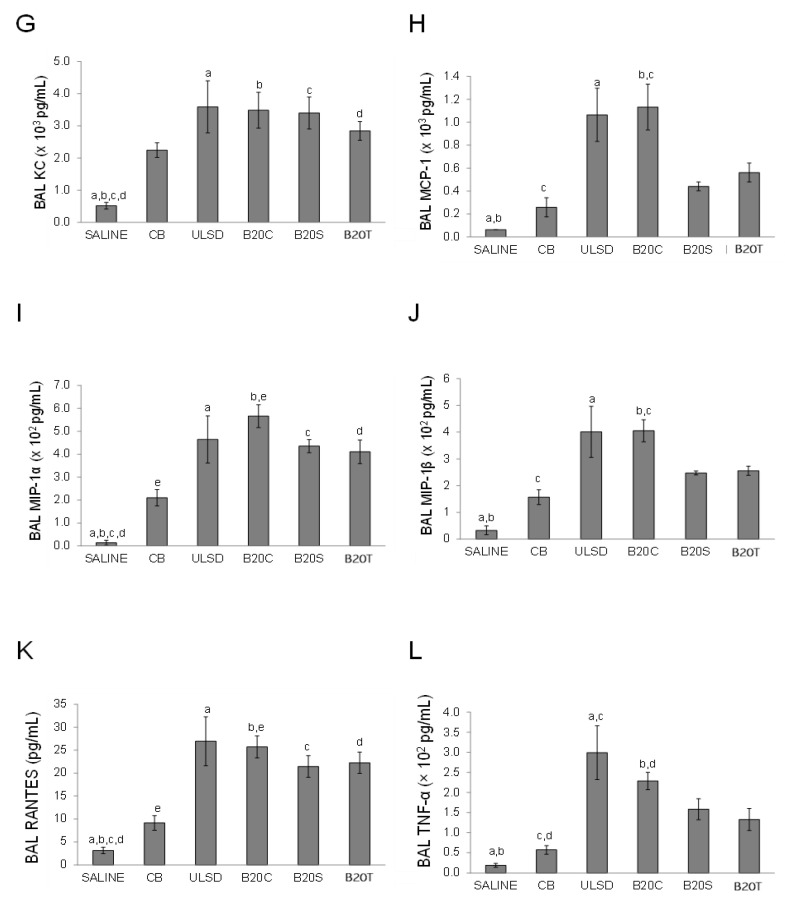
The figure shows the inflammatory cytokine (**A**–**L**) profiles in the BAL fluid following DEP exposures (values are mean ± SEM; n = 6/group; One-way ANOVA results: similar notations (letters) are significantly different (*p* < 0.05) from each other).

The levels of several inflammatory cytokines were also significantly increased in the bronchoalveolar lavage fluid in response to particle exposure in comparison to the saline vehicle control (one-way ANOVA, *p* < 0.05), with DEP_ULSD_ and DEP_B20C_ exposures showing increased cytokine levels in general compared to CB (Figure 4). 

In comparison to CB, instillation to DEPULSD significantly (*p* < 0.05) increased the levels of IL-6 and TNF-α (One-way ANOVA, *p* < 0.05, Figure 4D,L). Although the effects of DEPB20S or DEPB20T instillation were generally greater compared to the effects of CB instillation, the differences were not statistically significant. 

#### 3.2.3. Lung Gene Expression

**Figure 5:**
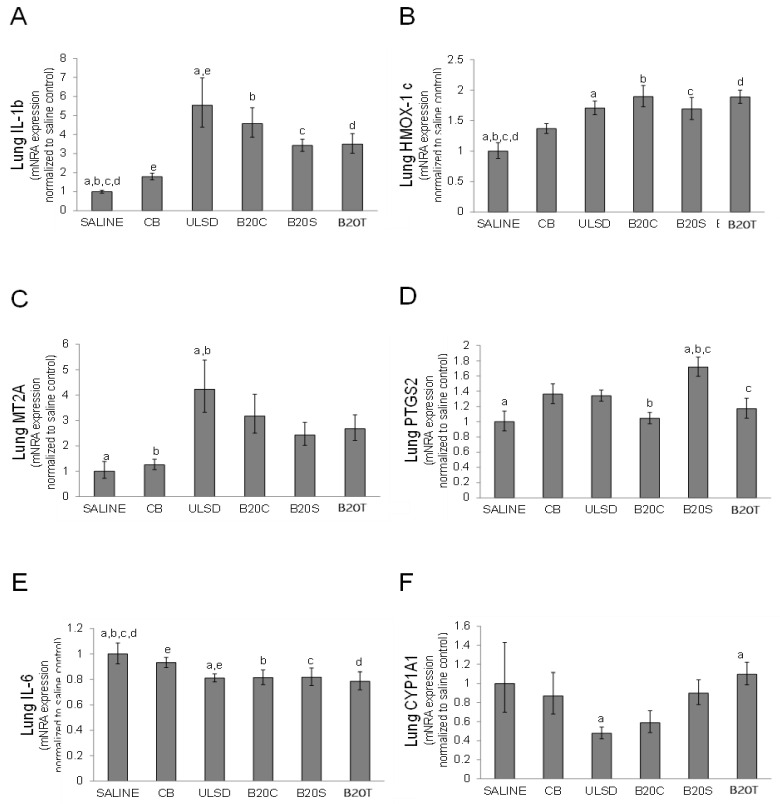
The figure shows the impact of DEP exposures on various gene expression (**A**–**F**) profiles in the lung tissue (values are mean fold change relative to time-matched control (saline) ± geometric standard deviation; n = 6/group; one-way ANOVA results: similar symbols (letter) are significantly different (*p* < 0.05) from each other).

In general, lung gene expressions after DEPULSD or DEPB20C exposures were comparable to responses of other DEPs (Figure 5). DEPULSD exposure led to significant increases in the expressions of lung IL-1β and MT2A and a significant decrease in IL-6 when compared to the effects of CB exposure (Figure 5A,C,E). Lung CYP1A1 was significantly decreased by exposure to DEPULSD in comparison to DEPB20T (one-way ANOVA, *p* < 0.05, Figure 5F). 

#### 3.2.4. Heart Gene Expression

**Figure 6:**
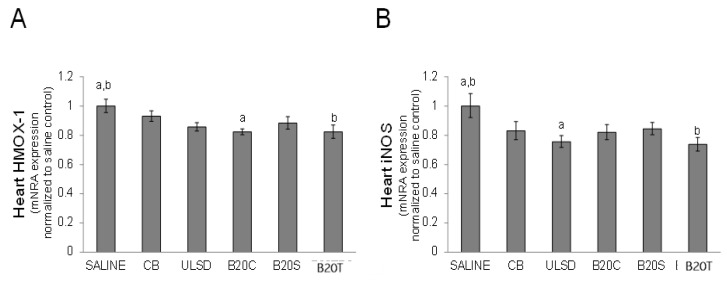
The figure shows the alteration of gene expression profiles for (**A**) HMOX-1 and (**B**)iNOS in the heart tissue following DEP exposures (values are mean fold change relative to time-matched control (saline) ± geometric standard deviation; n = 6/group; one-way ANOVA results: similar symbols (letters) are significantly different (*p* < 0.05) from each other).

The instillation of DEP significantly (*p* < 0.05) altered the expression of HMOX-1 and iNOS in the heart in comparison to saline, with the expression of HMOX-1 significantly decreased by exposure to DEP_B20C_ and DEP_B20T_ and the expression of iNOS significantly decreased by exposure to DEP_ULSD_ and DEP_B20T_ particles (One-way ANOVA, *p* < 0.05, Figure 6) compared to the vehicle control saline. 

#### 3.2.5. Plasma Cytokines

**Figure 7:**
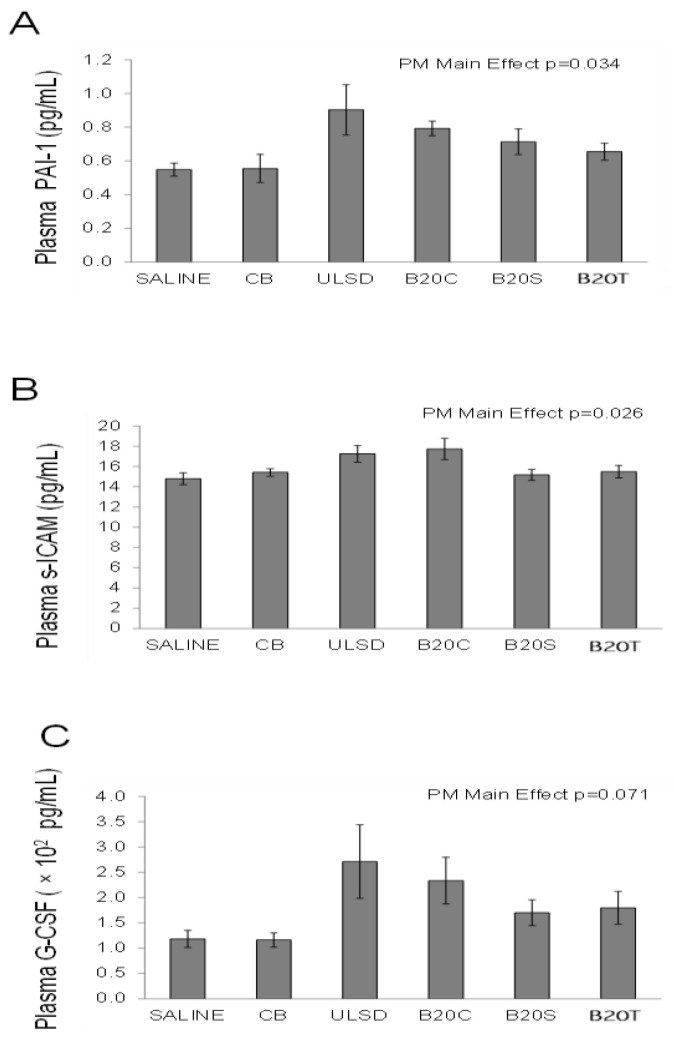
The figure shows the changes in plasma cytokine levels (**A**–**C**) following DEP exposures (values are mean fold change relative to time-matched control (saline) ± geometric standard deviation; n = 6/group; one-way ANOVA results: similar symbols (letters) are significantly different (*p* < 0.05) from each other).

Although the levels of PAI-1 and s-ICAM were impacted (Figure 7) by the treatments (one-way ANOVA, PM main effects, *p* < 0.05). The largest increases for PAI-1 and s-ICAM were observed after DEP_ULSD_ and DEP_B20C_ instillation. Although not statistically significant (*p* = 0.071), the levels of G-CSF were also increased by exposure to DEPs, with DEP_ULSD_ and DEP_B20C_ showing relatively higher responses compared to other DEPs. 

### 3.3. Correlations between In Vivo vs. In Vitro Toxicity Endpoints

In vitro cytotoxicity with secreted TNF-α in vitro (cellular ATP content, r = 0.93, *p* = 0.02; energy metabolism, r = 0.81, *p* = 0.1; LDH release, r= 0.82, *p* = 0.09). In vitro cytotoxic potency measured by examining the cellular ATP content, resazurin reduction (CTB), and LDH release correlated strongly (r > 0.9) and significantly (*p* < 0.05) with the levels of the inflammatory cytokines of G-CSF, IL-1a, IL-3, IL-6, IL-10, IL12(p40), MCP-1, MIP-1b, and TNF-α in the bronchoalveolar lavage fluid BAL (Table 4) after in vivo exposure. The cellular ATP level was significantly positively correlated with lung gene expressions for MT-2A, IL-1β and IL-6, while cellular CTB and LDH were negatively correlated with lung gene expression for CYP1A1. Cellular cytotoxicity endpoints were also positively correlated (*p* < 0.05) with extrapulmonary effects, including IL-1β gene expression in the heart and levels of MMP-9, G-CSF, PAI-1, s-ICAM, and s-VCAM in plasma. Similarly, positive correlations (*p* < 0.05) were observed between in vitro-secreted TNF-α levels and BAL cytokines, lung gene expressions (IL-1β and IL-6), heart gene expression (IL-1β), and plasma cytokine levels (G-CSF, PAI-1, and s-VCAM) 

## 4. Discussion

The selection of biodiesel blends used in this work was based on the fact that, at the time of testing, the major vegetable oils used in the production of biodiesels in the United States and Canada were soybean oil and canola oil. Another feedstock being used was animal tallow, and waste oils including grease. Our work showed engine load-related PM mass changes across all fuel types. We also noticed some differences in NOx levels due to engine load. Furthermore, the type of biodiesel blend influenced the emitted NOx levels (Table 3), which is consistent with previous reports about biodiesel source-dependent changes in NOx emissions [29,30]. Nevertheless, the focus of this work was to examine the relative toxicities of PM emitted when using different fuel types to support related health risk analysis. In this study, diesel exhaust particles from biodiesel blends (B20) with ULSD were examined for toxicity characteristics to understand the influence of feedstocks typically used in Canada on emitted particle toxicity. An effort was made to identify any consistency between the in vitro and in vivo toxicity behaviors of these particle emissions. Diesel exhaust particles mainly comprise two size modes, including fine particulate matter (PM2.5) based on our previous work [51] and are inhalable. Thus, our work here mainly focused on the pulmonary toxicity of these DEPs. For in vitro toxicity testing, the phagocytic mouse monocyte/macrophage cell type (J774A.1) was chosen since this cell type has been widely employed in in vitro pulmonary toxicity studies of PM, including engineered nanoparticles; in addition, the in vitro toxicity data from the use of this cell type will be suitable for correlation testing with the animal model used in this study to generate in vivo toxicity data. 

The in vitro cytotoxicity findings from this work showed that DEPs from ULSD and B20C consistently negatively affected all cytotoxicity endpoints more compared to other particle treatments, with greater responses seen in terms of cellular ATP (cell metabolism) and LDH (cell membrane integrity) contents. While most of the cell-secreted protein profiles showed main effects in terms of PM and dose, G-CSF and RANTES exhibited modifications to PM-exposure-related responses in relation to exposure dose. Carbon black appeared to be relatively less responsive compared to ULSD and the B20 biodiesel blends used in this work. Furthermore, it was interesting to note that the proinflammatory cytokine (TNF)-α profiles showed dose-related increases with particle exposures, while the anti-inflammatory IL-10 showed a decreasing trend with the dose of PM exposures, suggesting potential proinflammatory status in cells exposed to these particles. Paricularly, ULSD and B20C were more potent, followed by B20S and B20T, when compared to CB. The DEP of B20T was relatively less responsive among the biodiesel blends, and this may be attributed to its relatively higher saturated fat content compared to B20C and B20S that may have led to reduced carbonyl compound formation in particle emissions and, thus, relatively low cytotoxicity. Additionally, the fact that B20C is more potent than B20S in this work is in line with a report on mutagenicity testing conducted by Demarini et al., 2019 [52], where canola-based biodiesel emission particles were more potent than soy-based biodiesel emission particles. Furthermore, in vivo exposure to these DEPs and CB resulted in increased BAL neutrophils for all particle treatments compared to saline vehicle controls, and similarly secreted BALF proinflammatory cytokine levels were also increased for DEPs compared to the saline vehicle control group. Additionally, DEPs from ULSD and B20C exhibited relatively increased biological responses compared to CB and DEPs derived from B20S and B20T, which is consistent with in vitro toxicity findings in this work. 

Previous reports pointed to both the increased and decreased toxicity of combustion emissions from biodiesels or biodiesel blended fuels; the variance in the findings was generally due to the feedstock and blend ratios employed, engines and run cycles tested, and specific exhaust components (i.e., primary versus secondary particulate emissions, semi-volatile components, or whole exhaust) assessed. For example, Liu et al. 2008 [53] compared the cytotoxic potencies of particulate and semi-volatile constituents of diesel exhaust generated from the combustion of petroleum diesel or a palm methyl ester biodiesel blend at a range of blend ratios (10, 30, 50, 75, and 100%) in a four-stroke water-cooled, non-catalyst generator with a constant output power in BEAS-2B cells, and showed no significant differences in the toxicity of the particulate constituents between petroleum diesel and the biodiesel blends. However, the semi-volatile constituents from biodiesel exhaust were more toxic than those from petroleum diesel at all levels of blending. Likewise, the increased toxicity and heightened inflammatory response in cultured human epithelial cells exposed to whole exhaust from the combustion of a 20% *v*/*v* blend of canola biodiesel when compared to cells exposed to petroleum diesel exhaust, as reported by Mullins et al. 2014 [37] may, in part, relate to the toxicity of the gas phase that includes semi-volatiles. Using a four-stroke direct injection diesel engine from a tractor operated at a heavy-duty 13-mode test cycle ECE R49, Bunger et al., 2000 [54] showed that soot generated from the combustion of rapeseed biodiesel caused four-fold stronger toxicity in mouse fibroblasts when compared to soot from petroleum diesel. However, such load-dependent effects were only noted at ‘idling’ and not at the rated power. It was suggested that the higher toxicity was caused by carbonyl compounds and unburned rapeseed methyl ester-based fuel when the engine was idling. An integrated assessment of experimental factors such as engine type and load, biodiesel feedstock, blend ratios, batch-to-batch variability, and exposure to gas phase versus particulate phase versus whole exhaust will be critical to a holistic assessment of the automotive use of biodiesels derived from different feedstock. Our study only focused on the toxicity of particulate emissions, a key component of urban ambient airborne particulate matter that is implicated in a number of adverse population health outcomes. We combined particles derived from engine runs at 25% and 50% steady-state loads at a mass ratio of 2:1. While this maximized the sample mass available for our in vitro–in vivo comparisons, the mixing ratio also approximated the ratio of mass emission rates (g/bhp-hr) at the two steady-state loads for all the diesel exhaust particles (DEP_ULSD_, 1.81; DEP_B20C_, 1.94; DEP_B20S_, 1.96; and DEP_B20T_, 1.98), thereby giving equal weight to both loads in terms of mass emissions. 

The generally lower biological potency of primary particulate emissions derived from biodiesel blends than those derived from petroleum diesel in our study may relate to some of the previously documented decreases in the concentration of biologically reactive emission constituents with biodiesel blended fuels. In a comparison of particulate emissions from a compression ignition engine when using ultra-low sulfur diesel or blends of canola, soy, or tallow biodiesels with ultra-low sulfur diesel, Surawski et al. 2011 [30] showed that particle numbers and size distributions were feedstock-dependent, but the concentrations of particle and vapour phase PAHs, and reactive oxygen species were less sensitive to feedstock or blend percentage, and the concentrations were reduced with biodiesels when compared to ultra-low sulfur diesel. In a separate study, when a number of factors, such as nanoparticle size and number distribution, surface area distribution, elemental and organic carbon content, and polycyclic aromatic hydrocarbons adsorbed onto the particle surfaces were considered together to calculate toxic equivalency factors for B20 biodiesels of canola, soy, and animal tallow, it was adjudged that biodiesel particle toxicity was considerably lower in comparison to mineral diesel [55]. The study concluded that in spite of the higher PAH loading of emission particles from B20 combustion, their contribution to overall toxicity was less than that in diesel-derived particles. However, in the current work, although the in vitro toxicity of B20 blends of soy and animal tallow was relatively lower compared to ULSD, B20C (canola) exhibited cytotoxicity responses similar to that of ULSD. 

In this study, the in vitro assessment of DEPs focussed on the cytotoxicity and secretion of inflammatory cytokines and the in vivo assessment focused on a number of markers of inflammation, and cardiovascular effects; the effects were tested for correlation in an attempt to associate biological potency measured in vitro to in vivo effects related to potential pathophysiological outcomes of particle exposure. Several in vitro measures of toxicity based on cellular redox status, energy metabolism, and membrane integrity were well correlated to each other and to the in vitro secretion of the proinflammatory cytokine tumor necrosis factor (TNF)-α (Table 4). While it is known that in vitro exposure of cells to diesel exhaust particles elicits the secretion of inflammatory cytokines [56], noteworthy correlations were observed between cytotoxicity and inflammatory responses measured in vitro as well as in vivo. Acute inflammatory response to DEP exposure suggested by increased neutrophil extravasation in the lung was complemented by increased levels of the proinflammatory cytokines of IL-1α, IL-3, IL-6, IL-12(p40), MCP-1, and MIP-1 β in BALF. The increased gene expression of MT2A and the decreased expression of CYP1A1 mRNA in the lung with diesel particle exposure could be in response to the heightened inflammation status [57,58]. The instillation of diesel exhaust particles produced effects on the heart, with the increased gene expression of IL-1β, a proinflammatory cytokine, and decreased heart expression of heme oxygenase-1 (HMOX-1), an enzyme with an anti-inflammatory role. While increased plasma levels of G-CSF may also represent an inflammatory component, increased levels of the adhesion molecules s-ICAM may have cardiovascular consequences, especially in relation to its involvement in atherosclerotic processes [59]. The increased plasma concentration of plasminogen activation inhibitor (PAI)-1 in response to DEP exposure is also significant, as PAI-1 is known to play a significant role in fibrosis, a pathological formation of connective tissue [60]. 

The similarity of the ranking of in vitro and in vivo potency of DEP (ULSD ~ B20C > B20S > B20T) shows the potential utility and sufficiency of in vitro assays as screening assays for prioritizing environmental particles for animal studies and strengthens confidence in the validity of the toxicity ranking of the biodiesel emission particles. The use of in vitro assays for the toxicity screening of emissions arising from complex technology matrices consisting of engine types, fuels, run cycles, and after-treatment configurations in order to prioritize conditions for animal testing will be of considerable value in terms of cost, throughput, and reducing the use of animals. Our in vivo data show increased pulmonary inflammation, greater perturbation of cardiac gene expression, and changes in plasma markers relevant to the inflammation and cardiovascular effects relating to DEP in comparison to carbon black used as a surrogate of DEP with relatively low levels of organics, confirming that the effects are not simply a generalized response to particle exposure but rather are attributable to the composition and distinct properties of DEP. 

## 5. Conclusions

Both in vitro and in vivo analyses from the current work showed that biodiesel blending can decrease the biological potency of diesel exhaust particles based on the feedstock. The toxic potency of primary emission particles derived from the combustion of petroleum diesel (DEP_ULSD_) and the canola biodiesel–petroleum diesel blend (DEP_B20C_) were similar based on a number of in vitro and in vivo bioassays, while emission particles derived from the combustion of soy and tallow–waste oil biodiesel blends (DEP_B20S_ and DEP_B20T_, respectively) were less potent. Overall, our findings from the use of in vitro and in vivo PM exposure models suggest that the source of biodiesel can be an important determinant of DEP toxicity, and these results provide insight into potential underlying toxicity mechanisms, notably, inflammatory process. Advanced engine technologies and emission treatment technologies have significantly reduced gaseous and particulate emissions from new engines. Understanding the physicochemical determinants of toxicity with biodiesel blended fuels in newer engines needs to be considered in future toxicological studies to support the health risk assessment of these emissions. 

## Figures and Tables

**Table 1:** The table shows the engine specifications and certified emission rates.

Engine Manufacturer	Caterpillar
Model	C11
Year	2004
Serial number	KCA018109
Engine family	4CPXH0680EBK
Air handling system	Series Turbo-Charged
Control	Electronic ACERT
Bore (mm)	130
Stroke (mm)	140
Cycles	4
Number of cylinders	6
Displacement (liters)	11.1
Curb idle speed (rpm)	700
Rated test speed (rpm)	2100
Maximum torque (lb-ft)	1150 @ 1200 rpm
Maximum power (bhp)	305 @ 2100 rpm
Compression ratio	17.1
NOx (g/bhp-hr.) *	2.3
CO (g/bhp-hr.) *	1.6
PM (g/bhp-hr.) *	0.09

* Certified emissions with original DOC, when operated at 400HP at 1800 rpm, 1450 lb.-ft at 1200 rpm; data from https://www.epa.gov/compliance-and-fuel-economy-data/annual-certification-data-vehicles-engines-and-equipment (accessed on 6 April 2024).

**Table 2:** The table shows the analyses of the blended test fuels (B20).

Fuel Identification	Method	ULSD	Canola B20	Soy B20	Animal Tallow B20
Biodiesel blend Volume % *	ASTM D7371	n/a	19.6	19.3	20.2
Density, kg/m^3^ @ 15 °C	ASTM D4052	833.0	843.1	843.3	841.6
Cetane No. D613	ASTM D613	49.6	52.8	55.6	53.8
Carbon, %m	ASTM D5291	86.20	84.32	84.17	84.10
Hydrogen, %m	ASTM D5291	12.79	13.02	13.38	13.66
Sulphur, mg/kg	ASTM D5453	4.6	3.8	4.6	7.6

* Determination of biodiesel content in diesel fuel using mild infrared spectroscopy method by ARC.

**Table 3:** The table shows the engine operation conditions and emission rates.

Fuel	Engine Load	CO (g/bhp-hr.)	CO2 (g/bhp-hr.)	NOX (g/bhp-hr.)	THC (g/bhp-hr.)	PM (g/bhp-hr.)	FC (g/bhp-hr.)	Torque (lb.-ft)	Power (bhp)
ULSD	25%	0.07 (0.01)	559.8 (5.10)	1.73 (0.03)	0.023 (0.00)	0.107 (0.01)	177.3 (1.61)	561 (4.58)	129 (1.03)
	50%	0.04 (0.01)	492.6 (2.22)	1.63 (0.02)	0.004 (0.00)	0.059 (0.00)	156.0 (0.71)	844 (3.94)	194 (0.91)
B20 canola	25%	0.08 (0.02)	553.7 (0.96)	1.65 (0.02)	0.036 (0.00)	0.115 (0.00)	179.4 (0.31)	529 (1.29)	121 (0.31)
	50%	0.05 (0.01)	497.9 (6.21)	1.72 (0.02)	0.000 (0.00)	0.059 (0.00)	161.3 (2.02)	810 (8.63)	186 (1.98)
B20 Soy	25%	0.08 (0.02)	555.9 (1.23)	1.57 (0.01)	0.029 (0.00)	0.112 (0.00)	179.7 (0.40)	545 (0.60)	125 (0.13)
	50%	0.05 (0.01)	499.7 (2.07)	1.62 (0.01)	0.001 (0.00)	0.057 (0.00)	161.5 (0.67)	802 (2.98)	184 (0.64)
B20 Tallow	25%	0.06 (0.01)	566.2 (1.45)	1.78 (0.03)	0.028 (0.00)	0.115 (0.00)	183.0 (0.46)	534 (1.89)	122 (0.48)
	50%	0.04 (0.01)	504.0 (1.06)	1.71 (0.00)	0.007 (0.00)	0.058 (0.00)	162.9 (0.35)	799 (0.59)	183 (0.20)

Values in parentheses are standard deviations from n = 3 runs.

**Table 4:** The table show in vivo responses significantly correlated to a measure of cytotoxicity or secreted TNF-α in vitro: strength and significance of correlations *.

In Vivo Endpoints		In Vitro Cytotoxicity			In Vitro TNF-α Secretion
Compartment	Endpoint	ATP	CTB	LDH	
BAL cells	PMN Cell Number	0.792 (0.110)	0.688 (0.199)	0.739 (0.153)	0.774 (0.124)
BAL cytokines	G-CSF	**0.945** **(0.015)**	**0.919** **(0.027)**	**0.923** **(0.025)**	**0.909** **(0.032)**
	IL-1a	**0.964** **(0.008)**	0.850 (0.068)	0.871 (0.054)	**0.993** **(0.001)**
	IL-3	**0.901** **(0.037)**	0.708 (0.181)	0.756 (0.139)	**0.974** **(0.005)**
	IL-4	0.781 (0.119)	0.656 (0.230)	0.634 (0.251)	**0.947** **(0.014)**
	IL-6	**0.986** **(0.002)**	**0.953** **(0.012)**	**0.980** **(0.003)**	**0.889** **(0.044)**
	IL-10	0.842 (0.074)	**0.920** **(0.027)**	**0.888** **(0.044)**	**0.884** **(0.046)**
	IL-12(p40)	**0.921** **(0.026)**	**0.901** **(0.037)**	**0.913** **(0.030)**	**0.926** **(0.024)**
	MCP-1	**0.967** **(0.007)**	**0.915** **(0.029)**	**0.931** **(0.021)**	**0.931** **(0.021)**
	MIP-1α	0.821 (0.088)	0.631 (0.254)	0.652 (0.233)	**0.967** **(0.007)**
	MIP-1β	**0.983** **(0.003)**	**0.892** **(0.042)**	**0.921** **(0.026)**	**0.961** **(0.009)**
	KC	0.867 (0.057)	0.668 (0.217)	0.722 (0.169)	**0.920** **(0.027)**
	TNF-a	**0.972** **(0.006)**	0.850 (0.068)	**0.915** **(0.029)**	**0.890** **(0.043)**
Lung gene expression	CYP1A1	−0.867 (0.057)	**−0.966** **(0.008)**	**−0.952** **(0.012)**	−0.682 (0.204)
	MTII	**0.913** **(0.031)**	0.748 (0.146)	0.840 (0.075)	0.838 (0.076)
	IL-1β	**0.949** **(0.014)**	0.793 (0.109)	0.869 (0.056)	**0.897** **(0.039)**
	IL-6	**0.944** **(0.016)**	0.795 (0.108)	0.873 (0.053)	**0.879** **(0.050)**
Heart gene expression	IL-1β	**0.906** **(0.034)**	**0.922** **(0.026)**	**0.880** **(0.049)**	**0.913** **(0.030)**
Plasma cytokines	MMP-9	0.817 (0.091)	**0.913** **(0.030)**	**0.925** **(0.025)**	0.576 (0.310)
	G-CSF	**0.966** **(0.008)**	0.843 (0.073)	**0.909** **(0.033)**	**0.895** **(0.040)**
	PAI-1	**0.962** **(0.009)**	0.836 (0.078)	**0.904** **(0.035)**	**0.879** **(0.050)**
	s-ICAM	**0.911** **(0.031)**	**0.965** **(0.008)**	**0.943** **(0.016)**	0.831 (0.081)
	s-VCAM	**0.899** **(0.038)**	**0.895** **(0.040)**	0.863 (0.059)	**0.923** **(0.025)**

* Pearson product moment correlation; statistically significant correlations are highlighted in bold-faced font within brackets.

## Data Availability

Data is contained within the article or Supplementary Materials.

## Supplementary Materials

The following supporting information can be downloaded at: https://www.mdpi.com/article/10.3390/toxics12040290/s1, Table S1: Analyses of Unblended Biodiesels (B100); Table S2: Sample collection procedure, analytical methods, and instrumentation employed in the analyses of exhaust emissions during diesel exhaust particle sampling; Table S3: Primers used in the real-time PCR analysis of gene transcripts in the lung and heart tissues.

## References

[1] Hoffmann B., Moebus S., Möhlenkamp S., Stang A., Lehmann N., Dragano N., Schmermund A., Memmesheimer M., Mann K., Erbel R. (2007). Residential exposure to traffic is associated with coronary atherosclerosis. Circulation.

[2] Tonne C., Melly S., Mittleman M., Coull B., Goldberg R., Schwartz J. (2007). A case-control analysis of exposure to traffic and acute myocardial infarction. Environ. Health Perspect..

[3] Auchincloss A.H., Diez Roux A.V., Dvonch J.T., Brown P.L., Barr R.G., Daviglus M.L., Goff D.C., Kaufman J.D., O’Neill M.S. (2008). Associations between recent exposure to ambient fine particulate matter and blood pressure in the Multi-ethnic Study of Atherosclerosis (MESA). Environ. Health Perspect..

[4] Lall R., Ito K., Thurston G.D. (2011). Distributed lag analyses of daily hospital admissions and source-apportioned fine particle air pollution. Environ. Health Perspect..

[5] Raaschou-Nielsen O., Andersen Z.J., Jensen S.S., Ketzel M., Sørensen M., Hansen J., Loft S., Tjønneland A., Overvad K. (2012). Traffic air pollution and mortality from cardiovascular disease and all causes: A Danish cohort study. Environ. Health.

[6] Kirwa K., Eliot M.N., Wang Y., Adams M.A., Morgan C.G., Kerr J., Norman G.J., Eaton C.B., Allison M.A., Wellenius G.A. (2014). Residential proximity to major roadways and prevalent hypertension among postmenopausal women: Results from the Women’s Health Initiative San Diego Cohort. J. Am. Heart Assoc..

[7] Wichmann J., Sjöberg K., Tang L., Haeger-Eugensson M., Rosengren A., Andersson E.M., Barregard L., Sallsten G. (2014). The effect of secondary inorganic aerosols, soot and the geographical origin of air mass on acute myocardial infarction hospitalisations in Gothenburg, Sweden during 1985–2010: A case-crossover study. Environ. Health.

[8] Vincent R., Kumarathasan P., Goegan P., Bjarnason S.G., Guénette J., Karthikeyan S., Thomson E.M., Adamson I.Y., Watkinson W.P., Battistini B. (2022). Acute cardiovascular effects of inhaled ambient particulate matter: Chemical composition-related oxidative stress, endothelin-1, blood pressure, and ST-segment changes in Wistar rats. Chemosphere.

[9] Hazenkamp-von Arx M.E., Schindler C., Ragettli M.S., Künzli N., Braun-Fahrländer C., Liu L.-J.S. (2011). Impacts of highway traffic exhaust in alpine valleys on the respiratory health in adults: A cross-sectional study. Environ. Health.

[10] Delfino R.J., Wu J., Tjoa T., Gullesserian S.K., Nickerson B., Gillen D.L. (2014). Asthma morbidity and ambient air pollution: Effect modification by residential traffic-related air pollution. Epidemiology.

[11] Wilhelm M., Ritz B. (2003). Residential proximity to traffic and adverse birth outcomes in Los Angeles county, California, 1994–1996. Environ. Health Perspect..

[12] Yorifuji T., Naruse H., Kashima S., Ohki S., Murakoshi T., Takao S., Tsuda T., Doi H. (2011). Residential proximity to major roads and preterm births. Epidemiology.

[13] Mitku A.A., Zewotir T., North D., Jeena P., Asharam K., Muttoo S., Tularam H., Naidoo R.N. (2023). Impact of ambient air pollution exposure during pregnancy on adverse birth outcomes: Generalized structural equation modeling approach. BMC Public Health.

[14] Calderón-Garcidueñas L., Ayala A. (2022). Air Pollution, Ultrafine Particles, and Your Brain: Are Combustion Nanoparticle Emissions and Engineered Nanoparticles Causing Preventable Fatal Neurodegenerative Diseases and Common Neuropsychiatric Outcomes?. Environ. Sci. Technol..

[15] Raaschou-Nielsen O., Andersen Z.J., Hvidberg M., Jensen S.S., Ketzel M., Sørensen M., Loft S., Overvad K., Tjønneland A. (2011). Lung cancer incidence and long-term exposure to air pollution from traffic. Environ. Health Perspect..

[16] Hung L.-J., Chan T.-F., Wu C.-H., Chiu H.-F., Yang C.-Y. (2012). Traffic air pollution and risk of death from ovarian cancer in Taiwan: Fine particulate matter (PM2.5) as a proxy marker. J. Toxicol. Environ. Health A.

[17] Dummer T.J.B., Yu X., Cui Y., Nauta L., Saint-Jacques N., Sweeney Magee M., Rainham D.G.C. (2023). Traffic-Related Air Pollution and Risk of Lung, Breast, and Urinary Tract Cancer in Halifax, Nova Scotia. J. Occup. Environ. Med..

[18] Díaz-Robles L.A., Fu J.S., Reed G.D. (2008). Modeling and source apportionment of diesel particulate matter. Environ. Int..

[19] Brandt E.B., Kovacic M.B., Lee G.B., Gibson A.M., Acciani T.H., Le Cras T.D., Ryan P.H., Budelsky A.L., Khurana Hershey G.K. (2013). Diesel exhaust particle induction of IL-17A contributes to severe asthma. J. Allergy Clin. Immunol..

[20] Gordon C.J., Schladweiler M.C., Krantz T., King C., Kodavanti U.P. (2012). Cardiovascular and thermoregulatory responses of unrestrained rats exposed to filtered or unfiltered diesel exhaust. Inhal. Toxicol..

[21] Labranche N., El Khattabi C., Dewachter L., Dreyfuss C., Fontaine J., van de Borne P., Berkenboom G., Pochet S. (2012). Vascular oxidative stress induced by diesel exhaust microparticles: Synergism with hypertension. J. Cardiovasc. Pharmacol..

[22] Karthikeyan S., Thomson E.M., Kumarathasan P., Guénette J., Rosenblatt D., Chan T., Rideout G., Vincent R. (2013). Nitrogen dioxide and ultrafine particles dominate the biological effects of inhaled diesel exhaust treated by a catalyzed diesel particulate filter. Toxicol. Sci..

[23] Pöss J., Lorenz D., Werner C., Pavlikova V., Gensch C., Speer T., Alessandrini F., Berezowski V., Kuntz M., Mempel M. (2013). Diesel exhaust particles impair endothelial progenitor cells, compromise endothelial integrity, reduce neoangiogenesis, and increase atherogenesis in mice. Cardiovasc. Toxicol..

[24] Kim J.Y., Kim J.-H., Kim Y.-D., Seo J.H. (2022). Ultrafine Diesel Exhaust Particles Induce Apoptosis of Oligodendrocytes by Increasing Intracellular Reactive Oxygen Species through NADPH Oxidase Activation. Antioxidants.

[25] McCormick R.L. (2007). The impact of biodiesel on pollutant emissions and public health. Inhal. Toxicol..

[26] Fontaras G., Karavalakis G., Kousoulidou M., Ntziachristos L., Bakeas E., Stournas S., Samaras Z. (2010). Effects of low concentration biodiesel blends application on modern passenger cars. Part 2: Impact on carbonyl compound emissions. Environ. Pollut..

[27] Cahill T.M., Okamoto R.A. (2012). Emissions of acrolein and other aldehydes from biodiesel-fueled heavy-duty vehicles. Environ. Sci. Technol..

[28] George I.J., Hays M.D., Snow R., Faircloth J., George B.J., Long T., Baldauf R.W. (2014). Cold temperature and biodiesel fuel effects on speciated emissions of volatile organic compounds from diesel trucks. Environ. Sci. Technol..

[29] Fontaras G., Kousoulidou M., Karavalakis G., Tzamkiozis T., Pistikopoulos P., Ntziachristos L., Bakeas E., Stournas S., Samaras Z. (2010). Effects of low concentration biodiesel blend application on modern passenger cars. Part 1: Feedstock impact on regulated pollutants, fuel consumption and particle emissions. Environ. Pollut..

[30] Surawski N.C., Miljevic B., Ayoko G.A., Elbagir S., Stevanovic S., Fairfull-Smith K.E., Bottle S.E., Ristovski Z.D. (2011). Physicochemical characterization of particulate emissions from a compression ignition engine: The influence of biodiesel feedstock. Environ. Sci. Technol..

[31] Hajbabaei M., Johnson K.C., Okamoto R.A., Mitchell A., Pullman M., Durbin T.D. (2012). Evaluation of the impacts of biodiesel and second generation biofuels on NO(x) emissions for CARB diesel fuels. Environ. Sci. Technol..

[32] Karavalakis G., Gysel N., Schmitz D.A., Cho A.K., Sioutas C., Schauer J.J., Cocker D.R., Durbin T.D. (2017). Impact of biodiesel on regulated and unregulated emissions, and redox and proinflammatory properties of PM emitted from heavy-duty vehicles. Sci. Total Environ..

[33] Jalava P.I., Tapanainen M., Kuuspalo K., Markkanen A., Hakulinen P., Happo M.S., Pennanen A.S., Ihalainen M., Yli-Pirilä P., Makkonen U. (2010). Toxicological effects of emission particles from fossil- and biodiesel-fueled diesel engine with and without DOC/POC catalytic converter. Inhal. Toxicol..

[34] Hawley B., L’Orange C., Olsen D.B., Marchese A.J., Volckens J. (2014). Oxidative stress and aromatic hydrocarbon response of human bronchial epithelial cells exposed to petro- or biodiesel exhaust treated with a diesel particulate filter. Toxicol. Sci..

[35] Rouleau M., Egyed M., Taylor B., Chen J., Samaali M., Davignon D., Morneau G. (2013). Human health impacts of biodiesel use in on-road heavy duty diesel vehicles in Canada. Environ. Sci. Technol..

[36] Gerlofs-Nijland M.E., Totlandsdal A.I., Tzamkiozis T., Leseman D.L.A.C., Samaras Z., Låg M., Schwarze P., Ntziachristos L., Cassee F.R. (2013). Cell toxicity and oxidative potential of engine exhaust particles: Impact of using particulate filter or biodiesel fuel blend. Environ. Sci. Technol..

[37] Mullins B.J., Kicic A., Ling K.-M., Mead-Hunter R., Larcombe A.N. (2016). Biodiesel exhaust-induced cytotoxicity and proinflammatory mediator production in human airway epithelial cells. Environ. Toxicol..

[38] Agarwal A.K., Singh A.P., Gupta T., Agarwal R.A., Sharma N., Rajput P., Pandey S.K., Ateeq B. (2018). Mutagenicity and Cytotoxicity of Particulate Matter Emitted from Biodiesel-Fueled Engines. Environ. Sci. Technol..

[39] Yanamala N., Hatfield M.K., Farcas M.T., Schwegler-Berry D., Hummer J.A., Shurin M.R., Birch M.E., Gutkin D.W., Kisin E., Kagan V.E. (2013). Biodiesel versus diesel exposure: Enhanced pulmonary inflammation, oxidative stress, and differential morphological changes in the mouse lung. Toxicol. Appl. Pharmacol..

[40] Kisin E.R., Yanamala N., Farcas M.T., Gutkin D.W., Shurin M.R., Kagan V.E., Bugarski A.D., Shvedova A.A. (2015). Abnormalities in the male reproductive system after exposure to diesel and biodiesel blend. Environ. Mol. Mutagen..

[41] Fukagawa N.K., Li M., Poynter M.E., Palmer B.C., Parker E., Kasumba J., Holmén B.A. (2013). Soy biodiesel and petrodiesel emissions differ in size, chemical composition and stimulation of inflammatory responses in cells and animals. Environ. Sci. Technol..

[42] Douki T., Corbière C., Preterre D., Martin P.J., Lecureur V., André V., Landkocz Y., Pottier I., Keravec V., Fardel O. (2018). Comparative study of diesel and biodiesel exhausts on lung oxidative stress and genotoxicity in rats. Environ. Pollut..

[43] Gavett S.H., Wood C.E., Williams M.A., Cyphert J.M., Boykin E.H., Daniels M.J., Copeland L.B., King C., Krantz T.Q., Richards J.H. (2015). Soy biodiesel emissions have reduced inflammatory effects compared to diesel emissions in healthy and allergic mice. Inhal. Toxicol..

[44] Madden M.C. (2016). A paler shade of green? The toxicology of biodiesel emissions: Recent findings from studies with this alternative fuel. Biochim. Biophys. Acta.

[45] Nadeau D., Vincent R., Kumarathasan P., Brook J., Dufresne A. (1996). Cytotoxicity of ambient air particles to rat lung macrophages: Comparison of cellular and functional assays. Toxicol. In Vitro.

[46] Kumarathasan P., Breznan D., Das D., Salam M.A., Siddiqui Y., MacKinnon-Roy C., Guan J., de Silva N., Simard B., Vincent R. (2015). Cytotoxicity of carbon nanotube variants: A comparative in vitro exposure study with A549 epithelial and J774 macrophage cells. Nanotoxicology.

[47] Vincent R., Vu D., Hatch G., Poon R., Dreher K., Guénette J., Bjarnason S., Potvin M., Norwood J., McMullen E. (1996). Sensitivity of lungs of aging Fischer 344 rats to ozone: Assessment by bronchoalveolar lavage. Am. J. Physiol..

[48] Poon R., Nakai J., Yagminas A., Benoit F., Moir D., Chu I., Valli V.E. (2002). Subchronic toxicity of chloral hydrate on rats: A drinking water study. J. Appl. Toxicol..

[49] Thomson E.M., Vladisavljevic D., Mohottalage S., Kumarathasan P., Vincent R. (2013). Mapping acute systemic effects of inhaled particulate matter and ozone: Multiorgan gene expression and glucocorticoid activity. Toxicol. Sci..

[50] Vincent R., Goegan P., Johnson G., Brook J.R., Kumarathasan P., Bouthillier L., Burnett R.T. (1997). Regulation of promoter-CAT stress genes in HepG2 cells by suspensions of particles from ambient air. Fundam. Appl. Toxicol..

[51] Vincent R., Kumarathasan P., Goegan P., Bjarnason S.G., Guénette J., Bérubé D., Adamson I.Y., Desjardins S., Burnett R.T., Miller F.J. (2001). Inhalation Toxicology of Urban Ambient Particulate Matter: Acute Cardiovascular Effects in Rats. Res. Rep. Health Eff Inst..

[52] DeMarini D.M., Mutlu E., Warren S.H., King C., Gilmour M.I., Linak W.P. (2019). Mutagenicity emission factors of canola oil and waste vegetable oil biodiesel: Comparison to soy biodiesel. Mutat. Res. Genet. Toxicol. Environ. Mutagen..

[53] Liu Y.-Y., Lin T.-C., Wang Y.-J., Ho W.-L. (2008). Biological toxicities of emissions from an unmodified engine fueled with diesel and biodiesel blend. J. Environ. Sci. Health Part A Tox. Hazard. Subst. Environ. Eng..

[54] Bünger J., Krahl J., Baum K., Schröder O., Müller M., Westphal G., Ruhnau P., Schulz T.G., Hallier E. (2000). Cytotoxic and mutagenic effects, particle size and concentration analysis of diesel engine emissions using biodiesel and petrol diesel as fuel. Arch. Toxicol..

[55] Agarwal A.K., Gupta T., Dixit N., Shukla P.C. (2013). Assessment of toxic potential of primary and secondary particulates/aerosols from biodiesel vis-à-vis mineral diesel fuelled engine. Inhal. Toxicol..

[56] Schwarze P.E., Totlandsdal A.I., Låg M., Refsnes M., Holme J.A., Øvrevik J. (2013). Inflammation-related effects of diesel engine exhaust particles: Studies on lung cells in vitro. BioMed Res. Int..

[57] Inoue K., Takano H. (2013). Metallothionein as a negative regulator of pulmonary inflammation. Curr. Pharm. Biotechnol..

[58] Wright K., Morgan E.T. (1990). Transcriptional and post-transcriptional suppression of P450IIC11 and P450IIC12 by inflammation. FEBS Lett..

[59] Witkowska A.M., Borawska M.H. (2004). Soluble intercellular adhesion molecule-1 (sICAM-1): An overview. Eur. Cytokine Netw..

[60] Ghosh A.K., Vaughan D.E. (2012). PAI-1 in tissue fibrosis. J. Cell Physiol..

