# Supplementary material for: Concordance between In Vitro and In Vivo Relative Toxic Potencies of Diesel Exhaust Particles from Different Biodiesel Blends
Source: Toxics. 2024 Apr 16;12(4):290. doi: 10.3390/toxics12040290 (PMC11054440; doi:10.3390/toxics12040290)
Supplement: Supplementary file 1 [file toxics-12-00290-s001.zip › toxics-2930286-supplementary.pdf]

SUPPLEMENTARY DATA DESCRIPTION

Supplementary Table S1. Chemical analyses of B100 biodiesels.

Supplementary Table S2. Sample collection processes, analytical methodologies, and instrumentation used in the characterization of emissions

Supplementary Table S3. Primers used in the real-time PCR analysis of gene transcripts in the lung and heart tissues.

Supplementary Table S1. Analyses of Unblended Biodiesels (B100)

Fuel Identification	Method	Canola	Soy	Animal Tallow
Density, kg/m3 @ 15 °C	ASTM D4052	882.7	884.8	877.0
Cetane No. D613	ASTM D613	52.30	54.70	66.70
Carbon, %m	ASTM D5291	76.76	76.80	76.27
Hydrogen, %m	ASTM D5291	12.02	11.44	11.66
Viscosity @ 40 °C, (cSt)	ASTM D445	4.382	4.242	4.737
Sulphur, mg/kg	ASTM D5453	4.0	1.0	14.0
Total Nitrogen, mg/kg	ASTM D4629	9.600	2.570	75.580
Ash, Sulphated, Mass %	ASTM D874	0.000	0.001	0.002
Cloud Point, °C	ASTM D2500	-8	0	12
Copper Corrosion	ASTM D130	1a	1a	1a
Flash Point, °C	ASTM D93, Procedure A	136.0	130.0	167.0
Carbon Residue, Mass %	ASTM D4530	0.000	0.001	0.001
Acid Number mg KOH/g	ASTM D664	0.260	0.270	0.400
Free Glycerine, mass %	ASTM D6584	<0.001	<0.001	0.001
Total Glycerine, mass %	ASTM D6584	0.102	0.134	0.061
Monoglycerides, mass %	ASTM D6584	0.319	0.463	0.152
Diglycerides, mass %	ASTM D6584	0.095	0.081	0.120
Triglycerides, mass %	ASTM D6584	0.015	0.012	0.027
Water and Sediment, Volume %	ASTM D2709	Not tested	0.000	0.000
Inductively Coupled Argon Plasma	-	-	-	<0.001*
Group I metals (Na + K), mg/kg	EN 14107 modified	<1	-	-
Group II metals (Ca + Mg), mg/kg	EN 14107 modified	<1	-	-
Phosphorus, mg/kg	EN 14107 modified	<1	-	-
Calcium (Ca), mg/kg	ASDTM D5185 modified	-	<0.05	<0.05

Potassium (K), mg/kg	ASDTM D5185 modified	-	<0.05	<0.05
Magnesium (Mg), mg/kg	ASDTM D5185 modified	-	<0.05	<0.05
Sodium (Na), mg/kg	ASDTM D5185 modified	-	3.5	1.0
Phosphorus (P), mass %	ASDTM D5185 modified	-	<0.001	<0.001
	-			

Supplementary Table S2. Sample collection procedure, analytical methods, and instrumentation employed in the analyses of exhaust emissions during diesel exhaust particle sampling

Compound	Analysis Method	Instrument	Sample Collection
Carbon Monoxide (CO)	Non-Dispersive Infrared Detection (NDIR)	HORIBA Model AIA-210 LE	Continuous Collection
Carbon Dioxide (CO_2)	Non-Dispersive Infrared Detection (NDIR)	HORIBA Model OPE-115	Continuous Collection
Oxides of Nitrogen (NOx)	Heated Chemiluminescence Detection	California Analytical Instruments<br>Model 400-HCLD	Continuous Collection
Total Hydrocarbons (THC)	Heated Flame Ionization Detection (FID)	California Analytical Instruments<br>Model 300M-HFID	Continuous Collection
Particulate Matter<br>(PM)	Gravimetric Procedure	Sartorius<br>M5P-00V001	70mm Emfab Filters
Fuel Consumption (FC)	Calculated	Based on Carbon Balance	

Supplementary Table S3. Primers used in the real-time PCR analysis of gene transcripts in the lung and heart tissues.

Primer (Accession #)	Primer Sequence	Amplicon Size (bp)
TATA Binding Protein<br>TBP<br>(NM_013684.3)	Sense (22-): ccaatgactcctatgacccccta<br>Antisense (309-): cagccaagattcacggtagat	107
Oxyguanine glycosylase<br>OGG-1<br>(NM_010957.4)	Sense (348-): gccaacaaagaactgggaaa<br>Antisense (422-): aggtcagcactgaacagcac	95
Interleukin 1 beta<br>IL-1ß<br>(NM_008361.3)	Sense (86-): tgagcaccttcttttccttca<br>Antisense (171-): ttgtctaatgggaacgtcacac	104
Interleukin 6<br>IL-6<br>(NM_031168.1)	Sense (327-): gctaccaaactggatataatcagga<br>Antisense (381-): ccaggtagctatggtactccagaa	78
Endothelin<br>ET-1<br>(ENSMUST00000021796.6| ENSMUSG00000021367.6)	Sense (465-): ggacatcatctgggtcaaca<br>Antisense (568-): tgggaagtaagtctttcaaggaa	121
Inducible nitric oxide synthase<br>iNOS<br>(NM_010927.3)	Sense (2573-) : ccgatttagagtcttggtgaaag<br>Antisense (2646-): aatgtccaggaagtaggtgagg	95
Endothelial nitric synthase<br>eNOS<br>(ENSMUST00000030834.3| ENSMUSG00000028978.5)	Sense (2066-):tgatacgctatgcgggcta<br>Antisense (2133-): cagccatgttggatacagagc	88

		74
Cytochrome P4502, family 1, subfamily A, polypeptide 1<br>CYP1A1<br>(NM_001136059.1)	Sense (1103-): ttcctgtcctccgttacctg<br>Antisense (1155): tctgtgatgtcccggatgt	
Metallothionein 2A<br>MT2A<br>(NM_008630.2)	Sense (287-): ccgatctctcgtcgatcttc<br>Antisense (375-): caggagcaggatccatcg	107
Prostaglandin-endoperoxide synthase 2<br>PTGS2<br>(NM_011198.3)	Sense (100-): gtctctcaatgagtaccgcaaa<br>Antisense (154-): ctgcagccatttccttctct	75
Heme oxygenase-1<br>HO-1<br>(NM_010442.2)	Sense (250-): ggtcaggtgtccagagaagg<br>Antisense (321-): gcttgttgcgctctatctcc	91
Hypoxia-inducible factor<br>HIF-3α<br>(NM_016868.3)	Sense (86-): ttctgcaaggtcgacaactc<br>Antisense (171-): agcagcgagggagctagg	104
